# Parental and infant predictors of infant anger and fear reactivity

**DOI:** 10.3389/fpsyg.2026.1716579

**Published:** 2026-02-06

**Authors:** Laura Maria Wade-Bohleber, Agnes Von Wyl

**Affiliations:** 1 Institute for Mental Health, School of Applied Psychology, Zurich University of Applied Sciences, Zürich, Switzerland; 2 Department of Adult Psychiatry and Psychotherapy, University Hospital of Psychiatry Zurich, University of Zurich, Zürich, Switzerland

**Keywords:** anger, emotion regulation, fear, infancy, mind-mindedness, parental sensitivity

## Abstract

**Introduction:**

Infant regulation capacities result from a complex learning process influenced both by infant inborn dispositions and parental capacities for effective co-regulation. At the end of their first year of life, infants differ in their capacity to modulate anger and fear, two evolutionary adaptive emotions with important guiding functions for infant behavior.

**Aim:**

The aim of this pilot study was to elicit associations of anger and fear reactivity with parental mind-mindedness, parental sensitivity, and infant early regulation.

**Methods:**

In a sample of 47 families with infants aged 10.3 months on average, linear regression models served to test associations of variables of interest.

**Results:**

Higher maternal mind-mindedness predicted lower infant anger reactivity. Maternal sensitivity, mother rated infant early regulation, and paternal characteristics were not associated with infant anger reactivity. None of the considered parental and infant characteristics were associated with fear reactivity.

**Conclusion:**

Our findings provide preliminary evidence that the maternal capacity to interpret infant cues in terms of the underlying mental states is particularly important for the infant’s learning processes enabling the regulation of states of anger. The emotion-specific nature of this association (anger but not fear) suggests that different emotions may engage distinct co-regulatory processes, warranting further investigation. Our findings contribute to a growing body of research illustrating the complex relationships of parental mind-mindedness and parental sensitivity with infant emotion regulation.

## 1 Introduction

Infants acquire emotion regulation capacities during their first year of life through repeated experiences of co-regulation with their parents. We first describe the developmental process through which infants internalize parental co-regulation and discuss differences in the trajectories of the regulation of anger and fear, two evolutionary adaptive emotions with important guiding functions for infant behavior. We then review both infant contributions (early regulation dispositions) and parental contributions (sensitivity and mind-mindedness) to this co-regulatory process. Finally, we present the aims of the current pilot study, which investigates how these infant and parental characteristics predict anger and fear reactivity at the end of the first year of life.

### 1.1 Acquiring emotion regulation capacities during the first year of life: internalizing experiences of co-regulation

Humans are born with a surprising array of perceptual and learning abilities that enable them to engage in the social world around them (Stern, 1985). Only hours after birth, social contingencies between newborns and parents can be observed, typically when parents adjust their stimulation in gaze, voice, and touch to the newborn’s cues of distress (Feldman, 2012). Newborns have very limited capacities to regulate their own internal states and almost completely depend on their parents to fulfill their needs. Intuitively, parents mirror the affective states of the infant and the infant associates the marked affective states displayed by his or her parents with his or her inner states (Gergely and Watson, 1996; Markova and Nguyen, 2023). Gradually, the infant forms representations their inner states and internalizes the repeated experiences of parental co-regulation. This process allows the infant to develop regulatory strategies of his or her own and is part of the dyadic mutually regulating communication system between infant and parents (Tronick, 2017). The infant’s regulatory strategies become part of relatively stable patterns of behavior in the course of development (Rutter, 1989; Tronick and Beeghly, 2011).

At the end of the first year of life, the regulation of anger and fear seems of special interest for investigation regarding the development of infant emotional competencies. Anger and fear are both evolutionary adaptive emotions. Anger enables the infant to overcome obstacles that hinder his or her achievement of a goal (e.g., the goal to reach an interesting toy). Anger can thus be understood as an approach emotion that fosters the infant’s active engagement in the world (Lewis, 2010). Fear is an important emotion that helps the infants avoid dangers in their environment and to elicit protective behavior from his or her parents (e.g., when approached by a stranger) (Bowlby, 1969; Carver, 2004). While both emotions are adaptive when they can be flexibly regulated by infants and children, unregulated anger and fear have been associated with maladaptive social behaviors later in development (Eisenberg et al., 1994; Gilliom et al., 2002; Mills-Koonce et al., 2015).

Research indicates that infants and toddlers employ distinct regulation strategies depending on whether they experience fear or anger. In fear-eliciting contexts, infants and toddlers frequently use self-soothing, withdrawal, avoidance, and fussing to gain maternal attention, which can reduce fear by helping to escape threatening stimuli (Buss and Goldsmith, 1998; Diener and Mangelsdorf, 1999; Leerkes and Wong, 2012). In contrast, when experiencing anger or frustration, toddlers tend to adopt more complex regulatory techniques such as redirecting attention away from the source of distress, waiting passively, and actively seeking information—strategies that effectively diminish anger intensity (Cole et al., 2011; Gilliom et al., 2002). Evidence suggests that children predominantly use self-focused strategies when regulating fear, whereas they favor other-oriented approaches when managing anger (Ponzetti et al., 2023). How both parental and infant characteristics influence emotion-specific reactivity during the latter part of the first year remains largely unexplored.

### 1.2 Early infant contributions to co-regulation with parents

Infants take an active role within the mutually regulating communication system that unfolds during the first year of life. A broad body of research documents the different facets of social competencies in early infancy: By 3 months of age, infants actively engage in interactions with their parents and socially coordinate their behavior (Feldman, 2007). At 10 and 15 months, infants evaluate interactions based on fairness and expect positive or negative reciprocity in interactions (Jin et al., 2024; Meristo and Surian, 2013). In addition, research highlights the dependence of early social orientation on facial expressions. For instance, infants allocate more attention to fearful faces (Miguel et al., 2019; Peltola et al., 2008) and infants’ attention depends on maternal cues, as recently shown in a fairness distribution task (Geraci et al., 2025). Such early social competencies are likely antecedents of later prosocial behavior (Kuhlmeier et al., 2014). While the study of prosocial and moral development has yielded a broad and interdisciplinary body of research (Woo et al., 2022), the current investigation focuses specifically on the interplay of infant and parental contributions to infant affect reactivity.

The infants’ capacity to engage effectively in co-regulation is shaped not only by these early social competencies but also by individual differences in biological predispositions. Newborns differ in their inborn reactivity and regulation, two aspects of their temperament (Rothbart, 1981). For newborns perceived by their parents as highly reactive or irritable, establishing a functioning relationship of co-regulation may be more challenging (van den Bloom and Hoeksma, 1994). Adjusting to a highly reactive newborn, who typically has difficulties sleeping, feeding and soothing, can be very stressful for parents (Swain et al., 2011), although parental adjustment depends on different contributing factors such as spousal and social support, and parental mental impairments (Crockenberg, 1986; Feldman et al., 1997; Papousek and Papousek, 1990; Pauli-Pott et al., 2000). Overall, highly reactive infants are prone to experience more difficulties during their development (Degnan et al., 2008; Rothbart and Posner, 2006), and parental sensitivity may be even more crucial for their development (cf. differential susceptibility theory, Belsky, 2005) differential susceptibility theory. Given these differences in developmental trajectories, parental perception of infant regulation difficulties in the first weeks of life warrants investigation.

### 1.3 Parental contributions to co-regulation with their infant

Parents need to be capable of interacting sensitively with their infants to effectively co-regulate the infant’s states. Sensitive parents react in an appropriate and time-sensitive way to infant cues of distress (Ainsworth, 1979; Ainsworth et al., 1974). Sensitive parenting is the foundation for a secure attachment (Atkinson et al., 2000; De Wolff and van Ijzendoorn, 1997; Madigan et al., 2024), which is associated with more competent emotion regulation strategies than in insecure attachment classifications (Mikulincer et al., 2003) and predictive of various positive outcomes throughout development (Sroufe et al., 2005). Adult and child attachment are associated (van Ijzendoorn, 1995), yet parental sensitivity only partly mediates this relationship, a puzzling empirical observation described in the literature as the intergenerational transmission gap (van Ijzendoorn and Bakermans-Kranenburg, 2019). Biological dispositions and environmental stressors likely play a role in the intergenerational transmission gap as well as the parents’ mentalization. A recent meta-analyses illustrated that parental mentalization had a stronger predictive value for child attachment than parental sensitivity (Zeegers et al., 2017).

Parental mentalization refers to the parents’ capacity to form a representation of the internal states of their child and hold them in mind (Slade, 2005). Mind-mindedness, developed by Meins (1997), represents one operationalization of parental mentalization and can be understood as “reflective functioning in action”—the parent’s proclivity to spontaneously use their mentalizing capacity to describe and interpret the child’s behavior in terms of internal states. While parental mentalization describes the general capacity to mentalize, mind-mindedness specifically captures the tendency to apply this capacity spontaneously with the child. There is emerging empirical evidence supporting the association of parental mind-mindedness with parental affect co-regulation: Maternal mind-mindedness is related to mirroring behaviors in the Still Face Paradigm with five-months-old-infants (Bigelow et al., 2015). Interestingly, one study found no association of maternal mind-mindedness with infant negative affect during the Still Face Paradigm at 7 months (McMahon and Newey, 2018). However, a longitudinal study testing physiological markers of emotion regulation capacities (heart rate variability) over the first year of life demonstrated a clear link between parental mind-mindedness and infant emotion regulation (Zeegers et al., 2018).

Investigations of parent–child interactions and associations with child development have traditionally focused on mothers. However, a growing body of research explores the contributions of fathers to child development (Volling and Cabrera, 2019). Regarding parental mind-mindedness, Zeegers et al. (2018) demonstrated that paternal mind-mindedness links to physiological markers of infant emotion regulation, and Laflamme et al. (2022) showed that paternal mind-mindedness is associated with less parent-rated social–emotional problems. Despite these findings, the implications of paternal mind-mindedness for infant development remain understudied overall (McMahon and Bernier, 2017).

### 1.4 Aims of the current study

This pilot study aims to make a specific contribution to the existing literature by simultaneously examining both maternal and paternal mind-mindedness and sensitivity in relation to infant anger and fear reactivity at the end of the first year of life. By including both mothers and fathers and examining both representational (mind-mindedness) and behavioral (sensitivity) aspects of parenting alongside infant regulation difficulties in the first weeks of life, this study seeks to clarify which parental characteristics and infant characteristics are most predictive of anger versus fear reactivity at a critical developmental period. Further, we wanted to test the adequacy of our experimental set-up to examine these emotion-specific associations.

The following research questions guided the study:

Do maternal mind-mindedness and sensitivity and maternal rated infant early regulation predict infant anger reactivity at the end of first year of life? Do paternal mind-mindedness and sensitivity and paternal rated infant early regulation explain any more variance in infant anger reactivity than the maternal predictors alone?

Do maternal and paternal mind-mindedness and sensitivity and infant early regulation predict infant fear reactivity at the end of first year of life? Do paternal mind-mindedness and sensitivity and paternal rated infant early regulation explain any more variance in infant fear reactivity than the maternal predictors alone?

What insights do we gain for future studies concerning the experimental set-up chosen for this pilot-study?

We expected that maternal and paternal mind-mindedness and sensitivity would be negatively associated with both infant anger and fear reactivity. We assumed that infant early regulation would be positively associated with both infant anger and fear reactivity. However, given the functional differences between anger and fear, we explored whether the strength or pattern of these associations might differ between the two emotional contexts. Infant regulation capacities change with age (Braungart-Rieker et al., 2010; Calkins and Fox, 2002), which is why we controlled for infant age in our investigation.

## 2 Materials and methods

### 2.1 Participants

Fifty-one families were recruited through flyers, a university mailing list, internet platforms, and local daycare institutions. Inclusion criteria were infant age between 8 and 12 months and German or Swiss German as the family language. Exclusion criteria were an acute mental disorder of the parents, a premature birth (before pregnancy week 35), or a pervasive developmental disorder of the infant. Four families either chose not to continue with the study or did not respond to our contact efforts after the first home visit and dropped out from the study. The resulting final sample comprised 47 families. Table 1 summarizes the participants’ demographic characteristics. Overall, parents of our sample were highly educated (around 60% held a university degree) and rather older in age (mean age of 33.6 years (SD = 4.55) for mothers and 36.4 years (SD = 5.45) for fathers). Infants were on average 10.3 months old (SD = 1.36) and most infants were single children (over 60% had no siblings).

**Table 1:** Demographics of the sample.

Variables	Infants	Mothers	Fathers
Gender *n* (%)			
Female	25 (53.19)	47 (100)	
Male	22 (46.81)		47 (100)
No of siblings *n* (%)			
0	30 (63.83)		
1	14 (29.79)		
2	3 (6.38)		
Mean age (SD) [Min, Max]	Months: 10.3 (1.36) [8.18, 12.5]	Years: 33.6 (4.55) [25.5, 44.2]	Years: 36.4 (5.45) [27.6, 52.2]
Highest educational degree *n* (%)			
None		0 (0)	0 (0)
Compulsory elementary school		1 (2.13)	0 (0)
Vocational apprenticeship		5 (10.64)	8 (17.02)
Higher technical or vocational school		3 (6.38)	5 (10.64)
Gymnasium (highschool/college)		10 (21.28)	4 (8.51)
University		28 (59.57)	30 (63.83)
Current percentage of employment *n* (%)			
≤20%		7 (14.9)	1 (2.1)
≤40%		7 (14.9)	1 (2.1)
≤60%		13 (27.7)	2 (4.3)
≤80%		5 (10.6)	14 (29.8)
≤100%		0 (0)	29 (61.7)

SD, Standard Deviation.

All parents signed a written informed consent. The study was conducted in compliance with the university’s guidelines and Swiss legislation.

### 2.2 Procedures

Data were collected in two sessions of 30 to 45 min each. One session took place at the families’ homes and one in our research laboratory. The first session at the families’ homes consisted of a video-recorded free play interaction of each parent with the child and an audio-recorded interview, separately conducted with each parent. Additionally, parents filled in questionnaires. The second session in our research laboratory consisted of two video-recorded sequences of the Laboratory Temperament Assessment Battery (Lab-TAB, (Goldsmith and Rothbart, 1996)): the Stranger Approach and Gentle Arm Restraint. Before, in between, and after the Lab-TAB sequences, the mother and child were invited to play with each other without any instruction given. Only the infant and the mother participated in the second session. Infants received a little book as a gift at the end of the second session.

The decision to conduct the first session at home and the second in the laboratory was made for both practical and methodological reasons. Conducting the initial session at families’ homes reduced participant burden and facilitated recruitment by minimizing travel requirements, particularly important for families with infants. The home environment also provided a naturalistic setting for observing parent–child interactions and conducting interviews, which may elicit more spontaneous and representative parenting behaviors. The laboratory setting for the second session was chosen to ensure standardized conditions for the Lab-TAB procedures, which require controlled stimulus presentation and consistent environmental conditions to reliably assess infant affect reactivity. The laboratory environment also enabled high-quality video recording with fixed camera positions and optimal lighting, which is essential for accurate behavioral coding of infant emotional response, especially facial affect.

### 2.3 Instruments

#### 2.3.1 Infant characteristics

##### 2.3.1.1 Anger reactivity

Anger reactivity was assessed using the Gentle Arm Restraint sequence of the Lab-TAB (Goldsmith and Rothbart, 1996). In this sequence, the infant is seated in a highchair in front of a table. A colorful toy making different sounds and music is introduced to mother and infant and placed in front of them on the table. First, mother and infant can play with the toy. Once the infant is engaged with the toy, the mother gently holds back his arms so that he or she cannot reach the toy for 30s (gentle arm restraint episode 1). After this episode, mother and infant can play again with the toy. Then, the mother gently holds back the infant’s arms again for 30s (gentle arm restraint episode 2). After this, the infant can play again with the toy.

The Lab-TAB is a widely used experimental procedure in the field of temperament research and although the evidence is not extensive, existing studies suggest consistency over time in Lab-TAB based measures and moderate correlations with parental reports of temperament (Faísca et al., 2021; Gagne et al., 2011; Planalp et al., 2017).

The Lab-TAB Gentle Arm Restraint sequence was video recorded and rated by two psychologists using the software Interact (Mangold International GmbH, Germany) after extensive training. The two episodes of gentle arm restraint of 30s were subdivided into 5 s epochs. Each epoch was rated using the following codes:

(a) intensity of struggle: rating of the peak intensity of struggle on a 5-point Likert scale from 0 = no struggling at all, no resistance to 4 = high intensity struggle with continuous movement, moments of very high resistance;
(b) intensity of facial anger: rating of peak intensity of facial anger (using the AFFEX definition of facial expressions) on a 4-point Likert scale from 0 = no facial region shows codable anger movement to 3 = appearance in all 3 facial regions or strong facial anger;
(c) intensity of distress vocalizations: rating of peak intensity of stress vocalizations on a 6-point Likert scale from 0 = no distress to 5 = full intensity cry/scream. We calculated the mean of codes (a), (b), and (c) across epochs of the two episodes.

Ratings were conducted according to the Lab-TAB manual and more detailed information on the codes can be found in the manual. The two psychologists were trained using a test video of one infant. Seven cases were rated by both psychologists. ICCs indicated a good to excellent interrater reliability (Koo and Li, 2016) between the two raters: for (a) 0.88, 95% CI [0.68–0.97], for (b) 0.88, 95% CI [0.66–0.96] for (c) 0.78, 95% CI [0.42–0.93]. The remaining cases were split between the two psychologists to rate.

Following previous research using the Lab-TAB (Braungart-Rieker et al., 2010), we aggregated the individual anger component codes into a composite anger reactivity index. This approach is consistent with the conceptualization of anger reactivity as a multifaceted construct that encompasses facial, vocal, and bodily expressions of distress in response to frustrating situations. We calculated a mean across codes to form an overall anger reactivity score for further statistical analyses. Means of code (a), originally a 5-point Likert scale, and code (c), originally a 6-point Likert scale, were transformed to a 4-point Likert scale (by dividing means by 4 or 5, respectively, and multiplying them by 3) before calculating the mean score over all codes.

##### 2.3.1.2 Fear reactivity

Fear reactivity was assessed using the Stranger Approach sequence of the Lab-TAB (Goldsmith and Rothbart, 1996). In this sequence, the infant is seated in a highchair, slightly in front of the mother so that the mother is not visible without turning around. A male stranger enters the room and slowly approaches the infant. Eventually, the stranger picks the infant up and holds the infant briefly on his lap. The sequence is divided into 3 stages of 30 s which subdivide into 3 epochs of 10 s each.

The Stranger Approach sequence was video recorded. Using the software Interact (Mangold International GmbH, Germany), two psychologists rated each epoch using the following codes:

(a) intensity of facial fear: rating of peak intensity of facial fear (using the AFFEX definition of facial expressions) on a 4-point Likert scale from 0 = no facial region shows codable fear movement to 3 = appearance in all 3 facial regions or strong facial fear;
(b) intensity of distress vocalizations: rating of peak intensity of stress vocalizations on a 6-point Likert scale from 0 = no distress to 5 = full intensity cry/scream;
(c) intensity of bodily fear: rating of peak intensity of bodily fear on a 4-point Likert scale from 0 = no sign of bodily fear to 3 = freezing or trembling of the entire body;
(d) intensity of escape: rating of peak intensity of escape on a 4-point Likert scale from 0 = no escape behavior or social referencing to 3 = vigorous escape behavior involving intense-full body movements like arching back, twisting or leaning away, hitting, pushing.

We calculated the mean for (a), (b), (c), and (d) across all rated epochs.

Ratings were conducted according to the Lab-TAB manual after extensive training with a test case. Ten cases were rated by both raters. ICCs indicated a good to excellent interrater reliability (Koo and Li, 2016) between the two raters: for (a) 0.88, 95% CI [0.61–0.97], for (b) 0.96, 95% CI [0.86–0.99], for (c) 0.74, 95% CI [0.25–0.92], and for (d) 0.96, 95% CI [0.85–0.99]. ICC for (a), (b), (c), and (d) combined was 0.95, 95% CI [0.90–0.97]. The remaining cases were split between the two psychologists to rate.

In line with previous research (Braungart-Rieker et al., 2010), we created a composite fear reactivity index by aggregating individual behavioral codes. This aggregation approach is theoretically grounded in the understanding that fear reactivity manifests through coordinated responses across multiple behavioral domains, including facial expressions, distress vocalizations, escape behaviors, and bodily tension. We calculated the mean of the codes (a), (b), (c), and (d) to form an overall fear reactivity score for further statistical analyses. The code (b) was the only code rated on a 6-point Likert scale; all other codes were rated on 4-point Likert scales. We therefore transformed the mean of code (b) to a 0-to-3-point range (by dividing means by 5 and multiplying them by 3) before calculating the mean score over all codes.

##### 2.3.1.3 Infant early regulation

We used three questions to retrospectively assess how parents judged the infant’s regulation in the first 4 weeks of life. We asked – in comparison to other infants – how easy or difficult it was (1) to soothe, (2) to feed the infant, and (3) for the infant to sleep. Answers were given on a 5-point Likert scale from “much easier” (0) to “much more difficult” (4). We calculated a mean score of the three items for further analyses. Because of the small number of items, we used interitem correlation as index of internal consistency. Interitem correlation was 0.39 and within the range of 0.15–0.50 recommended by Clark and Watson (1995).

#### 2.3.2 Parental characteristics

##### 2.3.2.1 Sensitivity

Parental sensitivity was assessed using the CARE-Index (Crittenden, 2006). A 5-min free play sequence with mother–infant and father–infant was video recorded. Videos were rated according to the *CARE-Index Manual: Infancy* (Crittenden, 2006) by an independent coder who had been certified by P. Crittenden (2006) (reliable at Level II; mean correlation coefficient of 0.88). The same person rated all the videos. The CARE-Index assesses parental interactive behavior on three scales (sensitivity, control, and unresponsiveness) and the child’s interactive behavior on four scales (cooperation, compliance, difficult, and passivity). We used the parental sensitivity scale to assess sensitivity of both mothers and fathers. Scores range from 0 to 14 with higher scores indicating more sensitive interactive behavior.

##### 2.3.2.2 Mind-mindedness

Parental mind-mindedness was assessed in a separate interview with each parent (Meins and Fernyhough, 2010). Parents were invited to spontaneously describe their infant by answering the question: “How would you describe *<name of child>*?” Parents were informed that there are no right or wrong answers and encouraged to elaborate their description using neutral prompts such as “and what more…” or “what else can you think of.” A psychologist transcribed answers verbatim and coded them according to the mind-mindedness coding manual (Meins and Fernyhough, 2010). The coding was reviewed by a second psychologist, expert in the concepts of mind-mindedness and parental reflective functioning, and differences in the coding were discussed and resolved in consent. The mind-mindedness coding scheme identifies mind-related comments of the parents. Mind-related comments are comments that refer to the internal states of the infant such as what the infant may be thinking, experiencing, or feeling. Mind-related comments are distinguished from other comments referring to the child but not in terms of internal states. In the parental descriptions given of their infants, each attribute of the infant was assigned to one of four categories:

(a) mental attributes that refer to the mental life of the infant (mind-related),
(b) behavioral attributes that refer to the infant’s behavior such as games, activities, interactions,
(c) physical attributes that refer to the infant’s appearance or age,
(d) general attributes group all other comments of the parents that are not (a), (b), or (c).

For further analysis, we calculated the proportion of mental, i.e., mind-related, attributes compared to all attributes provided by the parents in their description of their infants. This resulted in a single mind-mindedness score for mothers and fathers ranging from 0 to 1, indicating 0 to 100% mind-related attributes in the description of their infants.

### 2.4 Statistical analyses

We conducted statistical analyses in R Studio (Version 2022.7.1.554; R Studio, PBC 2009–2022). We used ggplot2 (Wickham, 2016) to create illustrations of the data.

Descriptive statistics served to explore the sample’s demographic characteristics and the distribution of variables of interest. ICC estimates and their 95% confidence intervals of Lab-TAB observational ratings were calculated using the irr package (v0.84.1, Gamer, 2019) based on the mean ratings of the different subscales, consistency, and a 2-way mixed-effects model.

We employed linear regression models to examine the associations between parental characteristics (mind-mindedness and sensitivity), infant characteristics (infant early regulation) and infant affect reactivity outcomes (anger and fear reactivity). A hierarchical approach was chosen to evaluate the incremental contribution of single paternal variables beyond maternal variables, allowing us to test whether specific fathers’ characteristics explained additional variance in infant outcomes after accounting for mothers’ characteristics.

Prior to conducting regression analyses, all predictors were standardized (mean-centered and divided by their standard deviation). Linear regression assumptions were evaluated through visual inspection of diagnostic plots. Specifically, we examined: (1) linearity and homoscedasticity by inspecting residual vs. fitted value plots; (2) normality of residuals through Q-Q plots and histograms of standardized residuals. We only observed minor deviations from homoscedasticity and normality. Multicollinearity was assessed through variance inflation factors (VIF), with VIF < 5 considered acceptable. Multicollinearity was low; variance inflation factors (VIF) were < 2.87 for all predictors.

Two separate linear regression models were specified: one predicting infant anger reactivity and one predicting infant fear reactivity. Infant age (in months) was included as a control variable in all models based on prior research demonstrating that infant emotion regulation capacities change significantly with age during the first year of life (Braungart-Rieker et al., 2010; Calkins and Fox, 2002). Given our limited sample size, we employed an alternative models approach rather than fully cumulative hierarchical models to limit the number of predictors. Stage 1 included all maternal predictors (sensitivity, mind-mindedness, and maternal-rated infant early regulation) plus infant age as a control variable. Stages 2–4 each tested a different paternal predictor (sensitivity, mind-mindedness, and paternal-rated infant early regulation, respectively) while retaining all Stage 1 predictors. This approach allowed us to examine whether each specific paternal characteristic explained variance in infant affect reactivity beyond maternal characteristics. Each Δ*R*^2^ represents the unique contribution of that specific paternal predictor beyond maternal predictors.

Listwise deletion of cases with missing data on any variable resulted in a final sample of *N* = 41 for the anger reactivity models and *N* = 39 for the fear reactivity models.

For each regression model, we report multiple indices of effect size and model fit. Standardized regression coefficients (*β*) represent the effect size for individual predictors, indicating the change in the outcome variable (in standard deviation units) associated with a one standard deviation increase in the predictor. The R^2^ statistic quantifies the proportion of variance in the outcome explained by the model at each stage. Change in *R*^2^ (Δ*R*^2^) between stages indicates the incremental variance explained by adding a new predictor. F-tests evaluated the statistical significance of *R*^2^ at each stage and Δ*R*^2^ between stages for anger reactivity models. For all parameter estimates, we report 95% confidence intervals (CI) to convey estimation uncertainty. Confidence intervals that exclude zero indicate statistically significant effects at *α* = 0.05. The width of confidence intervals provides information about precision of estimation, with narrower intervals indicating more precise estimates.

A power analysis conducted using G*Power version 3.1.9.6 (Faul et al., 2009) indicated that *N* = 55 would be required to detect a medium effect size (*f^2^* = 0.15) in a multiple regression with five predictors (80% power, α = 0.05). Detecting a small effect size (*f^2^* = 0.06) would require *N* = 133. Our final sample sizes were insufficient to detect small effects; thus, our findings should be interpreted as exploratory results that can inform hypotheses and guide variable selection for future adequately-powered studies.

The R code for the described inferential statistical analyses is available in the supplemental materials.

## 3 Results

**Figure 1:**
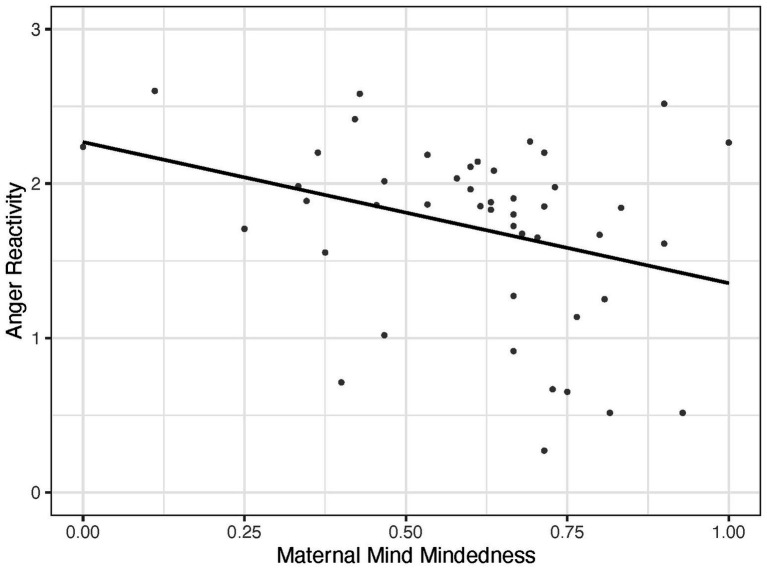
Association of maternal mind-mindedness with infant anger reactivity.

Regression analyses first tested associations of infant anger reactivity with maternal predictors (stage 1), and then associations of infant anger reactivity with paternal predictors in three consecutive stages. Regression results of stage 1 - 4 are reported in Table 2. Results of stage 1 indicated that maternal mind-mindedness, but neither maternal sensitivity nor mother rated infant early regulation were associated with infant anger reactivity R2 = 0.25, *F*(4, 36) = 3.01, *p* < 0.05. Higher levels of maternal mind-mindedness were associated with lower levels of infant anger reactivity as illustrated in Figure 1. Adding paternal sensitivity (stage 2), paternal mind-mindedness (stage 3), and paternal-rated infant early regulation (stage 4) did not significantly increase the explained variance of the model. Results of model comparisons are reported in the note of Table 2. Notably, while the addition of paternal sensitivity in Stage 2 did not result in a statistically significant increase in *R*^2^ (*p* = 0.07), the overall model remained significant (*F*(5, 35) = 3.29, p < 0.05). This indicates that paternal sensitivity contributes to the collective explanatory power of the model, even though its unique contribution beyond maternal predictors approaches but does not reach conventional significance. In contrast, adding paternal mind-mindedness (Stage 3) or paternal-rated infant early regulation (Stage 4) resulted in overall models that were no longer statistically significant (both *p* = 0.06).

**Table 2:** Results of linear regression predicting infant anger reactivity.

Predictors	Estimate	*SE*	95% CI *LL*	95% CI *UL*
Stage 1				
Intercept	1.70	0.08	1.53	1.87
Maternal predictors				
Sensitivity	0.13	0.09	−0.05	0.30
Mind-mindedness	−0.23	0.09	−0.42	−0.04
Infant early regulation	0.10	0.09	−0.07	0.28
Control variable				
Infant age	0.14	0.09	−0.04	0.32
*R*^2^ = 0.25, *F*(4, 36) = 3.01, *p* < 0.05				
Stage 2				
Intercept	1.70	0.08	1.53	1.86
Maternal predictors				
Sensitivity	0.10	0.09	−0.08	0.27
Mind-mindedness	−0.25	0.09	−0.44	−0.06
Infant early regulation	0.14	0.09	−0.04	0.32
Control variable				
Infant age	0.15	0.09	−0.02	0.33
Paternal predictor				
Sensitivity	0.17	0.09	−0.01	0.35
*R*^2^ = 0.32, *F*(5, 35) = 3.29, *p* < 0.05				
Stage 3				
Intercept	1.70	0.09	1.52	1.87
Maternal predictors				
Sensitivity	0.13	0.09	−0.05	0.31
Mind-mindedness	−0.23	0.10	−0.43	−0.04
Infant early regulation	0.11	0.09	−0.08	0.29
Control variable				
Infant age	0.14	0.09	−0.05	0.32
Paternal predictor				
Mind-mindedness	0.01	0.09	−0.17	0.19
*R*^2^ = 0.25, *F*(5, 35) = 2.35, *p* = 0.06				
Stage 4				
Intercept	1.70	0.09	1.52	1.87
Maternal predictors				
Sensitivity	0.12	0.09	−0.06	0.30
Mind-mindedness	−0.24	0.10	−0.43	−0.04
Infant early regulation	0.15	0.13	−0.11	0.41
Control variable				
Infant age	0.13	0.09	−0.05	0.32
Paternal predictor				
Infant early regulation	−0.06	0.12	−0.32	0.19
*R*^2^ = 0.26, *F*(5, 35) = 2.41, *p* = 0.06				

*N* = 41 for all stages. LL, lower limit; UL, upper limit. From stage 1 to stage 2: Δ*R*^2^ = 0.07, *F*(1, 35) = 3.56, *p* = 0.07, from stage 1 to stage 3: Δ*R*^2^ < 0.001, *F*(1, 35) = 0.02, *p* = 0.9, from stage 1 to stage 4: Δ*R*^2^ = 0.01, *F*(1, 35) = 0.26, *p* = 0.62.

Regression analysis revealed no associations of parental or infant characteristics with infant fear reactivity (cf. results of regression analysis stage 1–4 reported in Table 3).

**Table 3:** Results of linear regression predicting infant fear reactivity.

			95% CI	
Predictors	Estimate	*SE*	*LL*	*UL*
Stage 1				
Intercept	1.14	0.08	0.98	1.29
Maternal predictors				
Sensitivity	0.01	0.08	−0.15	0.17
Mind-mindedness	−0.04	0.08	−0.21	0.13
Infant early regulation	0.01	0.08	−0.14	0.17
Control variable				
Infant age	0.04	0.08	−0.12	0.20
*R*^2^ = 0.02, *F*(4, 34) = 0.17, *p* = 0.95				
Stage 2				
Intercept	1.14	0.08	0.98	1.30
Maternal predictors				
Sensitivity	0.01	0.08	−0.15	0.18
Mind-mindedness	−0.04	0.09	−0.21	0.14
Infant early regulation	0.00	0.08	−0.16	0.17
Control variable				
Infant age	0.03	0.08	−0.13	0.20
Paternal predictor				
Sensitivity	−0.04	0.08	−0.20	0.13
*R*^2^ = 0.03, *F*(5, 33) = 0.17, *p* = 0.97				
Stage 3				
Intercept	1.13	0.08	0.97	1.29
Maternal predictors				
Sensitivity	0.01	0.08	−0.15	0.18
Mind-mindedness	−0.05	0.08	−0.22	0.12
Infant early regulation	0.02	0.08	−0.14	0.18
Control variable				
Infant age	0.03	0.08	−0.13	0.19
Paternal predictor				
Mind-mindedness	0.06	0.08	−0.10	0.22
*R*^2^ = 0.04, *F*(5, 33) = 0.24, *p* = 0.94				
Stage 4				
Intercept	1.14	0.08	0.98	1.30
Maternal predictors				
Sensitivity	0.01	0.08	−0.16	0.17
Mind-mindedness	−0.04	0.09	−0.21	0.14
Infant early regulation	−0.03	0.11	−0.26	0.21
Control variable				
Infant age	0.04	0.08	−0.12	0.20
Paternal predictor				
Infant early regulation	0.05	0.12	−0.18	0.29
*R*^2^ = 0.03, *F*(5, 33) = 0.17, *p* = 0.97				

*N* = 39 for all stages. LL, lower limit, UL, upper limit.

As part of the Supplementary material, Supplementary Table S1 provides summary statistics of all variables of interest and Supplementary Figures S1, S2 graphically illustrate their distributions.

## 4 Discussion

The aim of this pilot study was to test associations of parental characteristics such as their mind-mindedness and sensitivity, and infant characteristics such as early regulation capacities with anger and fear reactivity at the end of the first year of life. We used experimental procedures to examine anger and fear reactivity in infants and narrative and observational procedures to capture the mind-mindedness and sensitivity of their parents. Parents also retrospectively rated the infant’s regulation capacities just after birth. An additional goal of this pilot study was to gain insights concerning the adequacy of the experimental design for future studies addressing similar research questions.

Parental and infant characteristics served to predict anger and fear reactivity in separate regression analyses. For anger reactivity and for fear reactivity, we first tested associations with maternal predictors (mind-mindedness, sensitivity, maternal-rated infant early regulation). Second, we tested if any of the paternal predictors (mind-mindedness, sensitivity, paternal-rated infant early regulation) would explain any more variance in infant anger and fear reactivity than maternal predictors alone.

We found that maternal mind-mindedness was negatively associated with infant anger reactivity. Neither maternal sensitivity nor mother rated infant early regulation was associated with infant anger reactivity. Paternal predictors did not explain any more variance in infant anger reactivity than maternal predictors alone. Regarding infant fear reactivity, we found no significant associations with either maternal or paternal mind-mindedness and sensitivity nor maternal or paternal rated infant early regulation. The pilot study illustrated specific advantages and disadvantages of the chosen experimental set-up, which we will discuss.

### 4.1 Maternal mind-mindedness is associated with infant anger reactivity

Our results indicate that infants of mothers who have a more pronounced tendency to think of their infants in terms of mental states show less anger reactivity. Reduced anger reactivity may be an indicator of more effective regulation at the end of their first year of life, although this can not be directly inferred from the measure used. In principle, this finding is in line with observations of how regulation capacities develop over the first year of life: Infants need their parents to function as external regulators of their internal states at first and they gradually internalize repeated experiences of parental co-regulation to develop regulatory strategies of their own. These processes are described in different theories in the literature, such as the social biofeedback model of parental affect-mirroring (Gergely and Watson, 1996), the Mutual Regulation Model (Tronick, 2017), the interactive psychobiological regulation processes highlighted by Schore and Schore (2008) and the role of parent-infant synchrony in the formation of affiliative bonds (Feldman, 2012) largely illustrated in empirical research (Beebe and Lachmann, 2020; Feldman, 2015). Our findings now extend this body of research by highlighting the importance of parental mind-mindedness for these early co-regulatory processes: The mother’s capacity to effectively regulate the infant’s states depends on her ability to interpret the infant’s cues in terms of the underlying needs by forming specific representations of the infant’s mental states, captured by the concept of mind-mindedness (Meins, 2013). Infants of a mother with adequate mind-mindedness are more likely to experience effective regulation and, in turn, to develop competent regulation. Our findings align with results reported from a longitudinal study showing that maternal mind-mindedness at 4 and 12 months predicts physiological markers of affect regulation capacities in infants at 12 months (Zeegers et al., 2018). A recent meta-analysis demonstrated the importance of parental mind-mindedness for child development beyond affect regulation: Parental mind-mindedness is associated with several positive developmental outcomes in the domain of executive functions, social cognition, and language (Aldrich et al., 2021). This is also consistent with the assumption that parent infant interactions provide the first context for the integration of self-regulation and social competency that both underly prosocial behavior and moral development (Schuetz and Koglin, 2023).

### 4.2 No association of maternal sensitivity with infant anger reactivity

While the literature provides solid evidence that maternal sensitivity influences infant socio-emotional development globally (Cooke et al., 2022; Deans, 2020) and infant emotion regulation specifically (Feldman, 2015) through direct and indirect pathways, our study did not find any association between parental sensitivity and infant anger or fear reactivity. We speculate that this may have to do with specific methodological aspects of how we assessed the two concepts. First, mind-mindedness assessed in an interview with the mother may have been a more robust measure of a maternal characteristic that fosters infant regulation than coding maternal sensitivity in a mother-infant free-play interaction. We hypothesize that mother-infant free-play interaction is more susceptible to the influence of context factors (e.g., how tired, hungry, fussy an infant or tired, preoccupied a mother is at that particular moment) than the description of the infant in an interview. There is some evidence illustrating that the expression of maternal sensitivity depends on the emotional context (Bozicevic et al., 2025; Leerkes et al., 2009). Future research should further clarify the role of contextual factors for the examination of parental sensitivity and mind-mindedness. Second, we know that crucial to infant development is not a “perfect” attunement of the parental response to the infants’ cues but rather that interactional mismatches can be repaired by parent and infant (Provenzi et al., 2018). In his mutual regulation model, Tronick (2017) describes parent-infant interaction that moves from being coordinated, matched and attuned to uncoordinated, mis-matched, and non-attuned and back. Repair is understood as the transition from a mismatch of affective states or behavior into matched states in the moment-to-moment interaction between parent and infant. The infant internalizes strategies of how to cope with these moments of mismatch: these are specific patterns of self-regulation that develop (Tronick and Gianino, 1986) and that likely contribute to emotion regulation abilities later on (Kemp et al., 2016). Parental mind-mindedness may be more predictive of these repair capacities in parent-infant dyads and a resulting effective co-regulation relationship than sensitivity ratings based on parent-infant-interactional behavior. In this sense, our findings may stipulate future research into the decisive parental contributions in the parent-infant interactional “dance” (Tronick, 2017). Third, our assessment of mind-mindedness may have captured more variance in parenting than our assessment of sensitivity in our sample, in which highly educated parents were over-represented. We used the CARE-Index (Crittenden, 2006) to assess parental sensitivity: Sensitivity scores range from 0–14 with 7 indicating a “normal” level of sensitivity and 14 an outstanding level of sensitivity. Scores of 6 and below indicate need for parental education or intervention. In our sample, only two mothers and four fathers had scores of 6 and below. In contrast, the distribution of scores in our mind-mindedness measures showed more variation. The CARE-Index was developed to work with parents compromised in their parenting abilities. It may be an instrument that is less sensitive to capture variations in parental sensitivity in a highly educated sample as ours. However, these are only speculations that need to be empirically studied in future research. Finally, the literature transmission gap describes the incomplete explanation of parental sensitivity and child attachment (van Ijzendoorn and Bakermans-Kranenburg, 2019; Zeegers et al., 2017). Child attachment correlates with strategies of emotion regulation (Obeldobel et al., 2023). It has been shown that parental mentalization is a stronger predictor of child attachment than parental sensitivity (Zeegers et al., 2017) and this may also be reflected in our findings.

### 4.3 No associations of paternal characteristics with infant anger reactivity

While paternal contributions to infant development remain overall understudied, some observations have linked paternal characteristics to infant regulation. Laflamme et al. (2022) showed that paternal mind-mindedness at 6 months predicted better infant adjustment at 18 months. In a study by Zeegers et al. (2018), not only maternal but also paternal mind-mindedness was associated with infant physiological markers of emotion regulation capacities at 12 months, an observation that does not match our results: We found no association of paternal mind-mindedness with infant reactivity at the end of the first year of life. This raises the question why maternal but not paternal mind-mindedness was related to infant anger reactivity in our study. We know that not only the quality (in terms of parental mind-mindedness or sensitivity) but also the quantity (involvement of parents) of parent–child interactions are important for infant development (Brown et al., 2012). Typically, fathers’ involvement with the child changes over time and is multi-determined (Planalp and Braungart-Rieker, 2016). In our study, we did not take into account the balance of maternal and paternal involvement in the infant’s upbringing. The demographic data of our sample only provides a hint that infants – on average – likely spent more day-to-day time with their mothers as fathers work at higher percentages than mothers at the end of the first year of the infant’s life. However, these are only speculations that need to be adressed in future research. Additionally, we assessed infant anger reactivity in presence of the mother and not the father, and we are curious if the same experimental procedure in presence of the father would have yielded different results in terms of infant anger reactivity and its association with paternal mind-mindedness. However, the distinct and mutual contributions of parental involvement and parental mind-mindedness to infant regulation capacities remain to be specified.

### 4.4 No associations of infant early regulation with infant anger reactivity

A surprising finding of our study was that parent rated infant early regulation was not associated with infant anger reactivity at the end of the first year of life. We relied on the retrospective rating of parents to capture differences in regulation in the first 4 weeks of life. Thus, we measured the parental perception of the early regulation dispositions of their child. Highly reactive infants at birth can make it more difficult for their parents to adjust and sensitively interact with them (van den Bloom and Hoeksma, 1994) and they are more prone to experience difficulties in their development (Degnan et al., 2008; Rothbart and Posner, 2006), possibly because it is more difficult for parents to establish a functioning relationship of co-regulation early on. Accordingly, we had expected different patterns of anger and fear reactivity at the end of the first year for infants who had been experienced as highly reactive or difficult in their temperament by their parents. Interestingly, only a small group of infants were rated by their parents as difficult in temperament than average (cf. Supplementary Figure S2). Further, one may speculate if the timing of our assessment was not appropriate to capture the contribution of infant inborn dispositions to the unfolding processes of co-regulation: At the end of the first year, differences in infant early regulation may have already been masked by parental regulation in the children of our sample. This would align with previous evidence demonstrating that specific types of parent-infant attachment are associated with different patterns of reactivity in infants who had been highly reactive at birth (Sherman et al., 2013). Finally, we relied on retrospective and parental ratings of infant early regulation, which may be biased by parents’ current perceptions of the child.

### 4.5 No associations of parental or infant characteristics with infant fear reactivity

Interestingly, we did not observe associations of parental mind-mindedness and sensitivity nor infant early regulation with fear reactivity. In our study, infant expressions of fear in face, body, and vocalization of infants during the Stranger Approach sequence were overall lower and showed less variance than the expression of anger in face, body, and vocalization during the Arm Restraint sequence (cf. Supplementary Figure S1). This may have contributed to the fact that we did not identify any associations with parental or infant characteristics. Our observed levels of fear reactivity were very similar to those reported in a study by Braungart-Rieker et al. (2010) for infants of the same age. These considerations raise important questions about the measurement sensitivity of the Lab-TAB Stranger Approach paradigm at 12 months when maternal presence is maintained. The lack of separation may create a ceiling effect, where the maternal buffer prevents most infants from displaying substantial fear responses, or alternatively a floor effect, where baseline fear levels are too low to detect meaningful individual differences. The restricted range of fear reactivity scores in our sample supports this interpretation and suggests that the paradigm may lack sufficient sensitivity to capture variance in fear responses under these conditions. Alternative experimental procedures that include actual separation from the caregiver (e.g., a modified Strange Situation) may be better suited to elicit and measure fear reactivity in 12-month-old infants, as they would create conditions more likely to produce observable individual differences in fear responses.

Beyond these methodological considerations, an additional consideration is that fear reactivity at 12 months may be more strongly influenced by stable temperamental dimensions that our current predictor variables do not capture. While parental mind-mindedness and sensitivity are theorized to shape emotion regulation through co-regulatory processes, fear responses—particularly in novel social situations—may reflect more biologically-based temperamental characteristics such as behavioral inhibition (Kagan, 2018). Further, there is emerging evidence that anger and fear follow distinct developmental trajectories and rely on different regulatory mechanisms during the first years of life (Diener and Mangelsdorf, 1999; Leerkes and Wong, 2012; Ponzetti et al., 2023). Research demonstrates that toddlers use other-oriented strategies to regulate anger and self-oriented strategies to regulate fear (Ponzetti et al., 2023). Anger, as an approach emotion aimed at overcoming obstacles to goals, appears to be particularly responsive to parental co-regulatory support, as infants naturally turn to caregivers for help when frustrated. In contrast, fear prompts self-protective responses where toddlers rely mainly on themselves, pulling away from threatening stimuli or using self-soothing behaviors. This emotion-specific pattern may also have implications in the sense that maternal mind-mindedness may be particularly crucial for anger regulation precisely because anger regulation relies more heavily on effective parent-infant co-regulation. However, these considerations remain speculative. Future research should explore whether mind-mindedness shows differential associations with anger versus fear across varied experimental contexts.

### 4.6 Insights concerning the experimental set-up

This pilot study provides important insights into the experimental set-up used that may be informative for the design of future studies. First, we used parental retrospective reports to assess infant early regulation. Retrospective parental report may be biased and future studies would ideally adopt a longitudinally approach and assess infant early regulation using observation-based measures. Second, we assessed parental mind-mindedness in an interview. Although it has been demonstrated that representational, i.e., interview, and interactional, i.e., play, assessments of mind-mindedness are equivalent (Aldrich et al., 2021), our use of the parental interview to assess mind-mindedness in our study had one important downside: We could not distinguish between appropriate and non-attuned mind-related comments, since parental descriptions could not be contrasted with direct observations oft the child. Recent evidence suggests that non-attuned mind-related comments have a specific predictive validity, for instance in predicting infant negative affectivity (McMahon and Newey, 2018) and this aligns with Meins (2013) assumption that appropriate and non-attuned comments do not represent two poles of the same continuum. Rather, while appropriate comments clearly relate to the concept of sensitivity, non-attuned comments may indicate a type of caregiving behavior that does not fit within the concept of sensitivity. Future studies on infant regulation should thus distinguish appropriate from non-attuned comments when collecting data on parental mind-mindedness. Third, we assessed anger and fear reactivity assuming that these would be indicators of infant self-regulation capacities at the end of the first year of life. However, the relationship between self-regulation and emotional reactivity at this age is complex and there are many different concepts describing and operationalizing these facets of infant emotions (Eisenberg et al., 2010). In the design of the current study, we did not differentiate regulation strategies and emotional reactivity, which limits the implications of our findings. Future studies could, for instance, consider how emotion regulation strategies change over the last months of the first year of life and how this affects anger and fear reactivity. Forth, while the fear and anger assessments using the Lab-TAB are well established (Planalp et al., 2017), other measures of emotional reactivity can be considered (Halle and Darling-Churchill, 2016). Especially, it is unclear, how ecologically valid these measures of emotional reactivity are as parents are instructed to not sensitively react to the infants distress during the procedure. Also, as discussed, fear reactivity may be measured best at the end of the first year of life with an actual separation of the mother or father. Fifth, it may be informative to more closely identify atypical cases in such a task—such as children with high anger reactivity and mothers with high mind-mindedness—and to describe the specific interactional patterns that characterize them.

### 4.7 Strengths and limitations

We designed this pilot study based on a thorough review of theoretical and empirical concepts of the development of infant emotion regulation during the first year of life. This study employed a methodically varied selection of instruments to assess relevant infant and parental characteristics: Infant anger and fear reactivity was tested in an extensive experimental procedure. Parental mind-mindedness and sensitivity measures were based on observer-rated interviews and videos of parent-infant interaction. Fathers were included in our investigation, and we thereby contribute to a growing body of research examining paternal as well as maternal contributions to children’s emotional development.

Several limitations of our study need to be addressed. First, the sample size of this pilot study with 47 families was relatively small. Our study was underpowered to detect small effect sizes. The sample size also limited the choice in statistical models. Ideally, the dyadic structure of our data would have been best captured by linear mixed models, which we were not able to employ because of the small sample size. Also, we employed an alternative models approach to test each paternal predictor separately rather than cumulatively, which prevented us from determining the unique contribution of each paternal variable when all are considered simultaneously. Further, other factors may contribute to infant fear and anger reactivity, such as breastfeeding (Drobetz et al., 2012). Also paternal engagement with the infant needs to be studied and there may be infant gender effects (Rouyer et al., 2007), which we also did not take into account here. Future studies should replicate our findings in a larger sample with enough power to detect small effect sizes, testing additional predictors, disentangle the independent contributions of multiple paternal characteristics, and employing statistical models that reflect dyadic data structure when working with observations for both mothers and fathers (Kenny et al., 2006). Second, highly educated parents were over-represented in our sample with around 60% of parents holding a university degree (compared to around 30% in the general population in Switzerland aged 25 to 44 years (Bundesamt für Statistik, 2022)). There is evidence that socioeconomic status is associated with parent-infant interaction and infant regulation (Gago-Galvagno and Elgier, 2020). A replication of our findings in an educationally and socioeconomically more diverse sample is thus also important. Third, we only collected cross-sectional data and could thus only test associations between variables.

## 5 Conclusion

As an exploratory pilot study, this research provides preliminary evidence and generates hypotheses for future investigation into the mechanisms linking parental characteristics to infant affect reactivity. We found that maternal mind-mindedness—the capacity to keep the infants’ internal mental states in mind—predicts anger reactivity at 12 months, while sensitivity measured by interaction observation does not. This finding contributes to the literature by identifying parental mentalization, operationalized by the concept of mind-mindedness, as a key mechanism through which co-regulation supports developing regulatory capacities, of which, in principle, infant reactivity can be an indicator. The emotion-specific nature of this association, whereby mind-mindedness predicted anger but not fear reactivity, aligns with emerging evidence that different emotions engage distinct regulatory processes during infancy, with anger regulation particularly dependent on parental co-regulatory support. Methodologically, our multi-method approach revealed important considerations for future research. The restricted range in sensitivity scores within our highly educated sample highlights the importance of measurement sensitivity to sample characteristics, while limited variance in fear reactivity when mothers remained present suggests alternative paradigms involving caregiver separation may better capture individual differences at this age. Future research should replicate these findings in larger, more diverse samples using longitudinal designs to examine how parental mind-mindedness shapes emotion regulation trajectories from birth into early childhood. Incorporating multiple measures of temperamental reactivity alongside parental mentalizing and parent-infant interaction would clarify the relative contributions of biological predispositions versus co-regulation processes.

## Data Availability

The datasets presented in this article are not readily available because of confidentiality constraints. The R code supporting the reported analyses is available in the supplementary material. Requests to access the datasets should be directed to laura.bohleber@zhaw.ch.
