# Supplementary material for: Parental and infant predictors of infant anger and fear reactivity
Source: Front Psychol. 2026 Feb 6;17:1716579. doi: 10.3389/fpsyg.2026.1716579 (PMC12920231; doi:10.3389/fpsyg.2026.1716579)
Supplement: Supplementary file 1 [file Table_1.DOCX]

**Table S1.** Summary Statistics of Variables of Interest.

|  | **N** | **Mean** | **SD** | **Median** | **Min** | **Max** |
| --- | --- | --- | --- | --- | --- | --- |
| Anger reactivity | 46 | 1.71 | 0.59 | 1.86 | 0.27 | 2.60 |
| Fear reactivity | 44 | 1.11 | 0.44 | 0.97 | 0.47 | 2.23 |
| Mind-mindedness (m) | 47 | 0.6 | 0.21 | 0.64 | 0 | 1 |
| Mind-mindedness (f) | 46 | 0.56 | 0.24 | 0.57 | 0 | 1 |
| Sensitivity (m) | 46 | 9.54 | 1.94 | 9.50 | 6 | 13 |
| Sensitivity (f) | 45 | 8.91 | 2.03 | 9.00 | 5 | 12 |
| Infant early regulation (m) | 46 | 1.58 | 0.96 | 1.67 | 0 | 3.67 |
| Infant early regulation (f) | 45 | 1.38 | 0.86 | 1.33 | 0 | 2.67 |

*Note*. SD = Standard Deviation, m = mothers, f = fathers.

**Figure S1.** Distribution of Anger and Fear Reactivity Scores.


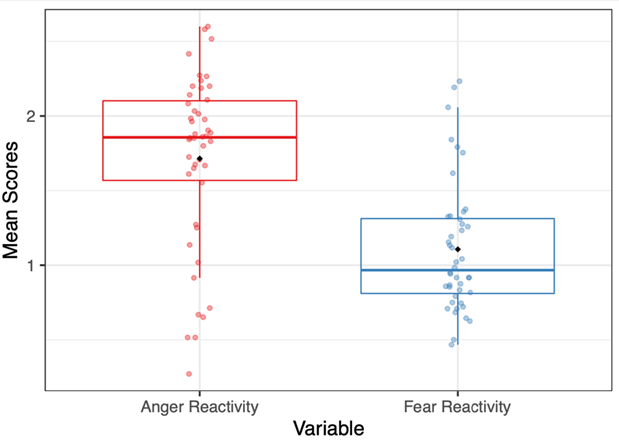


*Note.* Mean scores are indicated by a diamond. Higher scores indicate higher reactivity, with a minimum score of 0 and a maximum score of 3.

**Figure S2.** Distribution of Scores for Parental Sensitivity, Parental Mind-Mindedness, and Infant Early Regulation.


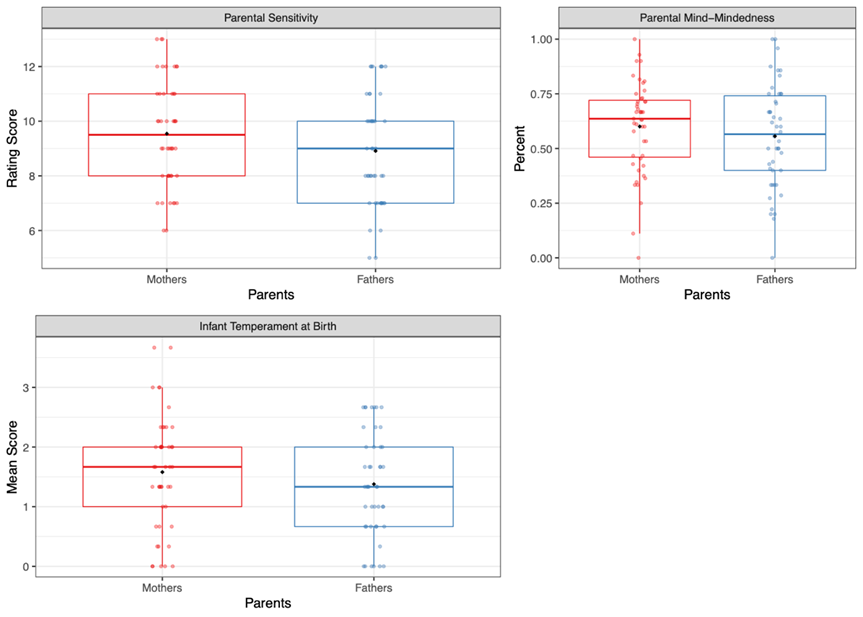


*Note.* Mean scores are indicated by a diamond. For parental sensitivity: higher scores indicate more sensitive parental behavior. For parental mind-mindedness: Percentage indicates the proportion of mind-related to not-mind-related comments. For infant early regulation: Higher scores indicate a more pronounced regulation difficulties (sleeping, feeding, soothing).

**R Code**

*Comment:*

*List of variables:*

*ci.s= care index parental sensitivity scale*

*mm = mind mindedness*

*temp = infant early regulation*

*m = mother*

*f = father*

*s = standardized*

###############################################################################/

#

# Regression analyses for anger reactivity -----

#

###############################################################################/

# exclude data where NAs for either mother or father

data.regr <- data1 %>%

# filter NA from analysis columns

dplyr::filter(!is.na(ci.s.m.s),

!is.na(mm.m.s),

!is.na(temp.m.s),

!is.na(age.child.s),

!is.na(ci.s.f.s),

!is.na(mm.f.s),

!is.na(temp.f.s)) %>%

# select relevant columns

dplyr::select(c('code','anger', 'fear',

'ci.s.m.s',

'mm.m.s',

'temp.m.s',

'age.child.s',

'ci.s.f.s',

'mm.f.s',

'temp.f.s'))

# regression model with maternal predictors

lm.angr.m <- lm(anger ~ ci.s.m.s + mm.m.s + temp.m.s + age.child.s, data = data.regr)

summary(lm.angr.m)

confint(lm.angr.m)

#Inspect distribution of residuals

plot(resid(lm.angr.m))

qqnorm(resid(lm.angr.m))

qqline(resid(lm.angr.m))

# check for multicollinearity of predictors

performance::check_collinearity(lm.angr.m)

# add paternal sensitivity to the model

lm.angr.m.f1 <- lm(anger ~ ci.s.m.s + mm.m.s + temp.m.s + age.child.s + ci.s.f.s, data = data.regr)

summary(lm.angr.m.f1)

confint(lm.angr.m.f1)

# compare models

anova(lm.angr.m, lm.angr.m.f1)

#Inspect distribution of residuals

plot(resid(lm.angr.m.f1))

qqnorm(resid(lm.angr.m.f1))

qqline(resid(lm.angr.m.f1))

# check for multicollinearity of predictors

performance::check_collinearity(lm.angr.m.f1)

# add paternal mind-mindedness to the model

lm.angr.m.f2 <- lm(anger ~ ci.s.m.s + mm.m.s + temp.m.s + age.child.s + mm.f.s, data = data.regr)

summary(lm.angr.m.f2)

confint(lm.angr.m.f2)

# compare models

anova(lm.angr.m, lm.angr.m.f2)

#Inspect distribution of residuals

plot(resid(lm.angr.m.f2))

qqnorm(resid(lm.angr.m.f2))

qqline(resid(lm.angr.m.f2))

# check for multicollinearity of predictors

performance::check_collinearity(lm.angr.m.f2)

# add paternal ratings of infant early regulation to the model

lm.angr.m.f3 <- lm(anger ~ ci.s.m.s + mm.m.s + temp.m.s + age.child.s + temp.f.s, data = data.regr)

summary(lm.angr.m.f3)

confint(lm.angr.m.f3)

# compare models

anova(lm.angr.m, lm.angr.m.f3)

#Inspect distribution of residuals

plot(resid(lm.angr.m.f3))

qqnorm(resid(lm.angr.m.f3))

qqline(resid(lm.angr.m.f3))

# check for multicollinearity of predictors

performance::check_collinearity(lm.angr.m.f3)

###############################################################################/

#

# Regression analyses for fear reactivity -----

#

###############################################################################/

# regression model with maternal predictors

lm.fear.m <- lm(fear ~ ci.s.m.s + mm.m.s + temp.m.s + age.child.s, data = data.regr)

summary(lm.fear.m)

confint(lm.fear.m)

#Inspect distribution of residuals

plot(resid(lm.fear.m))

qqnorm(resid(lm.fear.m))

qqline(resid(lm.fear.m))

# check for multicollinearity of predictors

performance::check_collinearity(lm.fear.m)

# add father sensitivity

lm.fear.m.f1 <- lm(fear ~ ci.s.m.s + mm.m.s + temp.m.s + age.child.s + ci.s.f.s, data = data.regr)

summary(lm.fear.m.f1)

confint(lm.fear.m.f1)

# compare models

anova(lm.fear.m, lm.fear.m.f1)

#Inspect distribution of residuals

plot(resid(lm.fear.m.f1))

qqnorm(resid(lm.fear.m.f1))

qqline(resid(lm.fear.m.f1))

# check for multicollinearity of predictors

performance::check_collinearity(lm.fear.m.f1)

# add father mind-mindedness

lm.fear.m.f2 <- lm(fear ~ ci.s.m.s + mm.m.s + temp.m.s + age.child.s + mm.f.s, data = data.regr)

summary(lm.fear.m.f2)

confint(lm.fear.m.f2)

# compare models

anova(lm.fear.m, lm.fear.m.f2)

#Inspect distribution of residuals

plot(resid(lm.fear.m.f2))

qqnorm(resid(lm.fear.m.f2))

qqline(resid(lm.fear.m.f2))

# check for multicollinearity of predictors

performance::check_collinearity(lm.fear.m.f2)

# add fathers' rating of infant early regulation (only)

lm.fear.m.f3 <- lm(fear ~ ci.s.m.s + mm.m.s + temp.m.s + age.child.s + temp.f.s, data = data.regr)

summary(lm.fear.m.f3)

confint(lm.fear.m.f3)

# compare models

anova(lm.fear.m, lm.fear.m.f3)

#Inspect distribution of residuals

plot(resid(lm.fear.m.f3))

qqnorm(resid(lm.fear.m.f3))

qqline(resid(lm.fear.m.f3))

# check for multicollinearity of predictors

performance::check_collinearity(lm.fear.m.f3)
